# Fabricating an Amperometric Cholesterol Biosensor by a Covalent Linkage between Poly(3-thiopheneacetic acid) and Cholesterol Oxidase

**DOI:** 10.3390/s90301794

**Published:** 2009-03-13

**Authors:** Po-Chin Nien, Po-Yen Chen, Kuo-Chuan Ho

**Affiliations:** 1 Department of Chemical Engineering, National Taiwan University/No. 1, Sec. 4, Roosevelt Rd., Taipei 10617, Taiwan; E-Mails: f94524006@ntu.edu.tw (P.C.N.); d94524009@ntu.edu.tw (P.Y.C.); 2 Institute of Polymer Science and Engineering, National Taiwan University/No. 1, Sec. 4, Roosevelt Rd., Taipei 10617, Taiwan.

**Keywords:** Cholesterol, cholesterol oxidase, covalent, ferrocene, poly(3-thiopheneacetic acid)

## Abstract

In this study, use of the covalent enzyme immobilization method was proposed to attach cholesterol oxidase (ChO) on a conducting polymer, poly(3-thiopheneacetic acid), [poly(3-TPAA)]. Three red-orange poly(3-TPAA) films, named electrodes A, B and C, were electropolymerized on a platinum electrode by applying a constant current of 1.5 mA, for 5, 20 and 100 s, respectively. Further, 1-ethyl-3-(3-dimethylamiopropyl)carbodiimide hydrochloride (EDC · HCl) and *N*-hydroxysuccinimide (NHS) were used to activate the free carboxylic groups of the conducting polymer. Afterwards, the amino groups of the cholesterol oxidase were linked on the activated groups to form peptide bonds. The best sensitivity obtained for electrode B is 4.49 mA M^−1^ cm^−2^, with a linear concentration ranging from 0 to 8 mM, which is suitable for the analysis of cholesterol in humans. The response time (t_95_) is between 70 and 90 s and the limit of detection is 0.42 mM, based on the signal to noise ratio equal to 3. The interference of species such as ascorbic acid and uric acid increased to 5.2 and 10.3% of the original current response, respectively, based on the current response of cholesterol (100%). With respect to the long-term stability, the sensing response retains 88% of the original current after 13 days.

## 1. Introduction

In clinical diagnosis, cholesterol is an important indicator in human blood for hypertension, myocardial infarction and arteriosclerosis. The normal range of total cholesterol in blood plasma, of which one-third is free cholesterol and two-thirds are cholesterol ester, is 3.96 ± 0.8 mM (153 ± 31 mg/100 mL) [1]. The value may be up to 8 mM for abnormal patients, and the high values have a relationship to the precursors of bile acid and steroid hormones. In previous studies, the sensing process is usually based on spectrophotometry [2,3]. However, the analysis involves complicated steps, hence, it is necessary to develop a sensing technique that may offer a faster response and higher selectivity. We have fabricated an amperometric biosensor by immobilizing an enzyme and this fulfills the above-mentioned requirements.

In general, enzyme-immobilization methods can be divided into four types; adsorption, entrapment, cross-linking and covalent attachment [4]. For the adsorption method, the enzyme is attached on the matrix either by hydrogen bonds or various charge attractions. For example, a multilayer of poly(2,6-pyridinedicarboxylic acid) with negative charge in the first layer and poly(allylamine hydrochloride) with positive charge in the second layer immobilizes negatively charged horseradish peroxidase by adsorption [5]. The entrapment process by electropolymerization in one-step, using conducting polymers, such as polypyrrole [6,7] and polyaniline [8], is a fast, convenient and popular method. During the entrapment process, the enzyme is entrapped inside the polymer and the thickness can be arbitrarily controlled by changing the polymerization time. In the cross-linking method, the use of a cross-linking agent such as glutaraldehyde [9] is necessary to link the enzyme and the substrate. However, in covalent attachment, chemical bonds are formed between the enzyme and the matrix through functional groups, such as amino or carboxylic groups. For instance, urease can be covalently immobilized on a copolymer film of poly(*N*-3-aminopropyl pyrrole) and polypyrrole, to fabricate an urea biosensor [10]; cholesterol oxidase (ChO) can be bound on a polyaniline-carbon nanotube composite film for a cholesterol biosensor [11]. Among the four methods mentioned, the covalent attachment and cross-linking methods provide better immobilizing performance than the others, which may be related to the presence of strong attractive forces formed by chemical bonds.

**Figure 1.**
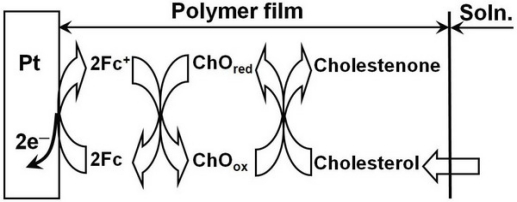
Schematic illustration of the sensing mechanism proposed for electrocatalytic oxidation of cholesterol on the modified electrode, where ferrocene acts as a mediator.

Judging by the previous literature, it was noted that functionalized conducting polymers such as polyazulenes [12] and polythiophenes [13,14] were proposed for the covalent attachment. In previous work on lactate biosensors [15], the carboxylic groups of poly(3-thiopheneacetic acid), which is one of the functional polythiophenes, were linked to the amino groups of lactate oxidase by two reagents, namely, 1-ethyl-3-(3-dimethylamiopropyl)carbodiimide hydrochloride and *N*-hydroxysuccinimide. In other words, the two chemicals are generally utilized as a carboxyl activating agent for providing a chemical bond with a primary amine group. Moreover, the physical properties and networks of poly(3-thiopheneacetic acid) obtained by electropolymerization were studied earlier [16]. Based on the above studies, an amperometric biosensor with poly(3-thiopheneacetic acid) as a matrix has been fabricated in this study for linking cholesterol oxidase, in order to carry out the detection of cholesterol. By using the conducting polymer in the linking method, the proposed biosensor may offer the advantage of better long-term stability. In addition, ferricinium ion (Fc^+^) was added as a mediator in the sensing system to prevent the formation of H_2_O_2_. The reaction sequence can be described by:

(1) $$\text{Cholesterol}+{\text{ChO}}_{\text{ox}}\to \text{Cholestenone}+{\text{ChO}}_{\text{red}}$$

(2) $${\text{ChO}}_{\text{red}}+{2\text{Fc}}^{+}\to {\text{ChO}}_{\text{ox}}+2\text{Fc}$$

(3) $$2\text{Fc}\to {2\text{Fc}}^{+}+{2e}^{-}$$

where ChO_ox_ and ChO_red_ represent the oxidized state and the reduced state of cholesterol oxidase, respectively. As the reactions involved are in series, the cholesterol concentration can be determined indirectly by sensing the current contributed from the electrochemical oxidation of Fc. The schematic illustration of the sensing mechanism in the polymer film during sensing is shown in Figure 1.

## 2. Results and Discussion

### 2.1. Electropolymerization of Poly(3-TPAA) Film

**Figure 2.**
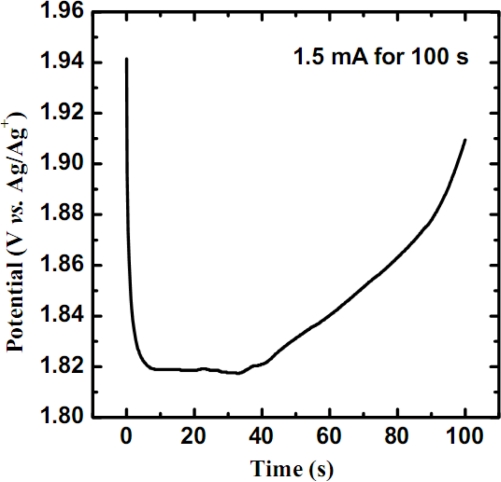
The chronopotentiometry of the poly(3-TPAA) film electroploymerized at a constant current of 1.5 mA for 100 s in an organic electrolyte.

The electropolymerization of poly(3-thiopheneacetic acid) film on a bare platinum electrode at a constant current is shown in Figure 2. The chronopotentiometric curve can be divided into three stages. In the first stage (*ca.* 0 ∼ 5 s), the monomer forms a dimer or radical species since the high potential would overcome the energy barrier. With the increase of radical ions, the potential (more precisely, the driving force, or the overpotential of the electrochemical reaction) appears to decrease and this implies that the deposition process was governed by a steady-state chain reaction. The polymer thin film was found to grow up stably at *ca.* 1.82 V (*vs*. Ag/Ag^+^) in the second stage (*ca.* 5 ∼ 40 s). Finally in the last stage (*ca.* 40 ∼ 100 s), the potential became large again. This is because the modified polymer film was too thick and resulted in high resistances during the electropolymerization. Besides, the brown polymer film was brittle and displayed swelling properties.

### 2.2. FTIR Spectrum, SEM Morphology and EIS Analysis

**Figure 3.**
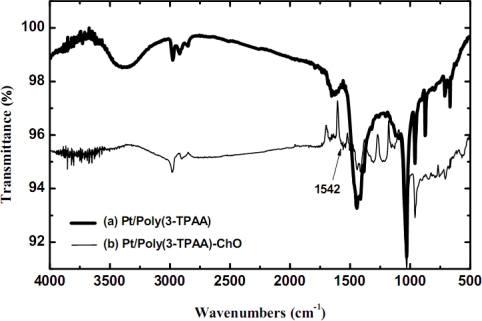
The FTIR spectra of Pt/poly(3-TPAA) film (bold line) and Pt/poly(3-TPAA)-ChO film (thin line) at room temperature.

Figure 3 shows the FTIR spectra of the Pt/poly(3-TPAA) electrode with and without cholesterol oxidase. In both spectra, the broad absorption at 3,200–3,500 cm^−1^ is assigned to the O-H of carboxylic groups and the absorption peaks at 2,977, 2,920 and 2,847 cm^−1^ resulted from aliphatic C-H groups. The aromatic ring stretches cause the peak at 1,350–1,550 cm^−1^ and the peak at 1,625 cm^−1^ may be due to the severe oxidation of the carboxylic groups of the polymer film to form esters during electropolymerization [16].

Comparing the two electrodes with (Figure 3, curve b) and without (Figure 3, curve a) the linking enzyme, the additional peak between 1,520–1,550 cm^−1^ in Pt/poly(3-TPAA)-ChO (which represents the Pt/poly(3-TPPA) electrode linked with cholesterol oxidase) film is assigned to the N-H bend of the cholesterol oxidase, which confirms the formation of covalent bonds between enzyme and polymer. Besides, the O-H of carboxylic groups (3,200–3,500 cm^−1^) vanished in Figure 3 (curve b) because the carboxylic groups were linked with the enzyme.

**Figure 4.**
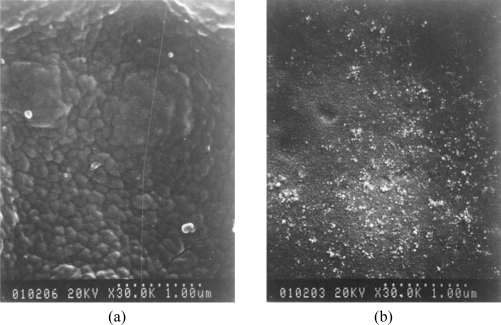
The SEM pictures of **(a)** Pt/poly(3-TPAA) and **(b)** Pt/poly(3-TPAA)-ChO.

The surface morphology of the electrode with and without cholesterol oxidase was monitored by scanning electron microscopy [Figures 4 (a) and (b)] at a fixed magnification of 30.0 K. Platinum was sputtered on the indium tin oxide (ITO) glass and used as the substrate. The electropolymerization condition and the covalent process were the same as mentioned earlier. The molecular weight of cholesterol oxidase is about 200 kD, which is approximately equal to the size of 10 nm. The enzyme appears as white particles in Figure 4(b), which clearly distinguishes the morphologies between the two electrodes and this also confirms the formation of covalent bonds between enzyme and polymer.

**Figure 5.**
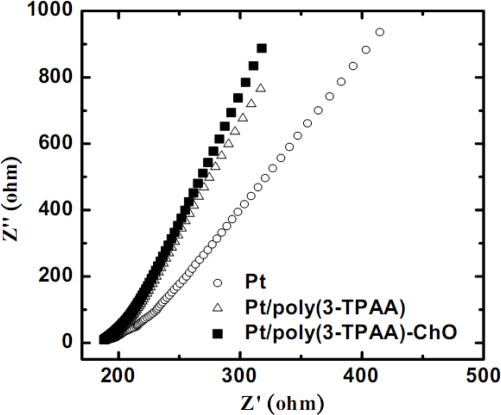
The electrochemical impedance spectroscopy data for the Pt, Pt/poly(3-TPAA) and Pt/poly(3-TPAA)-ChO electrodes in 0.1 M PBS with 1.0 mM Fe(CN)_6_^3−^.

Figure 5 is the electrochemical impedance spectroscopy of the electrodes measured at the open circuit potential with the frequency ranging from 65,000 to 400 Hz in 0.1 M PBS with 1.0 mM Fe(CN)_6_^3−^. The order of the slope, from low to high, is Pt, Pt/poly(3-TPAA) and Pt/poly(3-TPAA)-ChO. This means that the charge transfer resistance (R_ct_) at the Pt/poly(3-TPAA) was much larger than that of the bare Pt, but slightly smaller than that of the Pt/poly(3-TPAA)-ChO. This is because only a little enzyme was linked on the polymer, thus slightly increases the R_ct_ of the electrode. In short, the EIS analysis provides another proof that the ChO was indeed linked on the polymer.

### 2.3. Limiting Current Plateau and Electron Transfer

**Figure 6.**
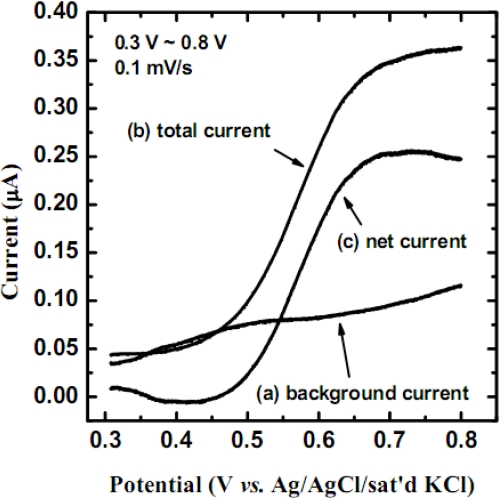
The LSV of electrode C scanned from 0.30 to 0.80 V (*vs*. Ag/AgCl/sat’d KCl) in background electrolyte (a) and 8 mM cholesterol solution (b), at a scan rate of 0.1 mV/s.

In order to determine the best sensing potential, the linear sweeping voltammetry (LSV) was performed for electrode C at low scan rate, as shown in Figure 6.

The total current and the background current were obtained respectively in the 8 mM cholesterol-containing solution and the background electrolyte described in Section 3.2. The total current was found to be higher than the background current. The net current, obtained from the total current minus the background current, is contributed from the oxidation of ferrocene. From the net current noted in Figure 6, it follows that the current responses can be divided into three parts. In the first part, the current approaches zero value in the potential range of 0.30 ∼ 0.45 V. This is due to the fact that ferrocene could not be oxidized on the electrode [Equation (3)] at lower potentials. At higher potentials, a sharp increase in the sensing current was noticed in the potential range of 0.45 ∼ 0.65 V, which supports the reaction sequence proposed in Equations (1) ∼ (3). Finally, in the potential range of 0.65 ∼ 0.80 V, the sensing current reaches a plateau, which is the limiting current region of the electrochemical reaction. The plateau observed in Figure 6 corresponds to the oxidation of ferrocene, as described by Equation (3). A potential of 0.70 V (*vs.* Ag/AgCl/sat’d KCl) was set as the best sensing potential for detecting cholesterol. Theoretically, the standard potential for ferrocene oxidation, E^0^, is 0.15 V (*vs.* Ag/AgCl/sat'd KCl). Yet, the potential to reach the limiting current plateau (0.65 ∼ 0.80 V) is higher in this study, which is common (or natural property) for an electrode modified with conducting polymer films. The net current, obtained by the LSV with no mediator (not shown), was smaller than the net current in Figure 6 (curve c) and no plateau was observed. This implied that the plateau of the catalyzed product (H_2_O_2_) would appear at potential exceeding 0.80 V, as the natural property of poly(3-TPAA) may shift the oxidation potential to more positive direction.

For the sake of obtaining the stoichiometric number of electron transfer involved in the electrochemical oxidation [Equation (3)], the following equation derived from the Nernst equation was applied:,

(4) $$E=E_{1/2}+\frac{\mathrm{RT}}{\mathrm{nF}}\text{ln}\left[\frac{I_{\infty }-I}{I}\right]$$

where *E* is the applied potential, *E_1/2_* is the half-wave potential at which the current has reached exactly half of its limiting current, *I_∞_* is the limiting current, *I* is the current at the applied potential, *E*, *n* is the number of electrons involved in the redox reaction, *R* is the gas constant equaling to 8.3144 J/mol · K, *F* is the Faraday constant equaling to 96,485 C/mol, and *T* is the temperature in Kelvin equaling to 298 at room temperture. According to Equation (4), a plot of log[(I_∞_-I)/I] vs. E is a straight line with a slope of n/59.1, from which n is calculated to be 0.79 in the potential range of 0.50 ∼ 0.60 V. The n value is taken approximately as unity and the deviation may come from the two chemical reactions (Equations (1) and (2)) proceed before the electrochemical reaction (Equation (3)).

### 2.4. The Calibration Curve of the Cholesterol and the Performances of the Biosensor

**Figure 7.**
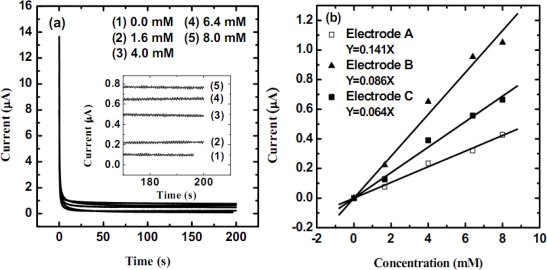
(a) The current responses of the cholesterol at different concentrations by applying the sensing potential at 0.70 V (*vs.* Ag/AgCl/sat’d KCl) on electrode C and (b) the calibration curves of the three modified cholesterol biosensors at 0.70 V with regressions.

Figure 7(a) reveals that the current responses of cholesterol at each concentration level do reach the steady-state values with a sampling time of 200 s. The sensing current increases with the increase in cholesterol concentration up to 8 mM. The calibration curves of the three cholesterol biosensors, as shown in Figure 7(b), were plotted from the steady-state current at 200 s for each cholesterol concentration. From Figure 7(b), it is seen that the sensing current is proportional to the cholesterol concentration and the linear range for all enzyme electrodes lies between 0 and 8 mM (the normal cholesterol range of human beings is 3.96 ± 0.8 mM). Among the three preparation times, too little functional group of cholesterol oxidase was linked on electrode A. By increasing the deposition time, the film became thicker and the sensitivity increased for electrode B. However, the sensitivity of electrode C decreased significantly due to the increase in resistance for a thick film. The best sensitivity obtained was 4.49 mA M^−1^, which was calculated from the slope of the regression line with a regression coefficient of 0.981. Furthermore, two additional performance parameters, such as the response time and the limit of detection (LOD), were obtained. The response time (t_95_) is defined as the time it takes for the electrode to reach 95% of the steady-state current (variant disturbance ≤20 nA), which was calculated to be 70 ∼ 90 s at different concentration levels. The LOD (with the signal to noise ratio of 3) was 0.42 mM. Besides, the Michaels-Menten constant of electrode B was calculated to be 14.53 mM with a R^2^=0.992 based on the calibration curve.

The interference effect with respect to the common species in human plasma, such as ascorbic acid and uric acid, has been examined. The normal ranges of ascorbic acid and uric acid are 0.04 ∼ 0.08 mM and 0.18 ∼ 0.42 mM, respectively. In this work, the tested concentrations of ascorbic acid and uric acid were chosen to be 0.10 and 0.40 mM, respectively, as determined by the upper limits in the blood. The net current response for cholesterol was set as 100%, and the relative percentages of the current increment in the presence of ascorbic acid and uric acid were 5.2 and 10.3%, respectively. It is interesting to note that the interference effect is not significant.

### 2.5. The Long-term Stability and Discussion on the Sensor Performance

**Figure 8.**
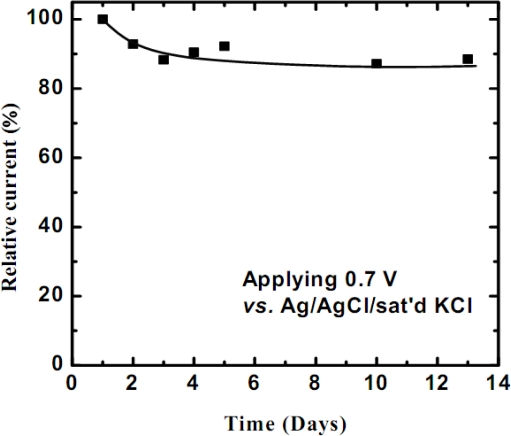
The long-term stability of the cholesterol biosensor at a sensing potential of 0.70 V *vs.* Ag/AgCl/sat’d KCl.

The covalent method provides an interactive binding force between the cholesterol oxidase and poly(3-TPAA) film. The long-term stability of the cholesterol biosensor is shown in Figure 7. By defining the sensing current collected at the first day as 100%, the current response contributed from cholesterol retained 88% of its original value after 13 days. The initial decrease in the sensing current may result from the loss of the enzyme, which was entrapped in the polymer matrix. During the daily analysis, it is possible that the loss of enzyme begins initially as the polymer chains are in motioning and swelling conditions. According to the long-term stability data in Figure 8, the biosensor achieves a stable condition in 4 ∼ 5 days. When comparing the performance of this cholesterol biosensor with those found in literatures (Table 1), this biosensor shows a wide linear range. The first seven references in Table 1 reported the data on the immobilization of an enzyme, ChO, with different enzyme electrodes to sense free cholesterol by the entrapment method. The next three reported the results using two kinds of enzymes, namely ChO and cholesterol esterase (ChEt), to detect the total cholesterol concentration in plasma. In the latter methods, the cholesterol ester is initially catalyzed by ChEt to form free cholesterol. The free cholesterol then reacted with another enzyme, ChO, to produce species following the reaction sequence described in Equations (1) ∼ (2). It is apparent that the performance of cholesterol biosensors, especially the enzyme-based sensors, depends on the kind of electrode substrate, matrix layer, and process used. The linear range, sensitivity and response time of this work are comparable with the others; however, the enzyme immobilization based on the covalent attachment provides the modified electrode with a better long-term stability than the entrapment method reported in literatures. Conversely, the LOD (limit of detection) in our system needs to be improved and this can be attained by using a flow injection system to reduce both the noise and the diffusion layer thickness, which form the basis of our future work.

## 3. Experimental

### 3.1. Chemicals and Instruments

Cholesterol oxidase (ChO) (EC 1, 1, 3, 6) from *Pseudomonas fluorescens* (protein approx. 15%), cholesterol (>99.0%), phosphate buffer saline (PBS, pH 7.4), potassium chloride (99.0 ∼ 100.5%) and the surfactant, Triton^®^ X-100 (for molecular biology), were purchased from Sigma. The monomer, 3-thiopheneacetic acid (3-TPAA), and the mediator, ferrocene, were obtained from Acros. The two linking agents, 1-ethyl-3-(3-dimethylamiopropyl) carbodiimide hydrochloride (EDC · HCl) and *N*-hydroxysuccinimide (NHS, >97.0%), were obtained from Sigma and Fluka, respectively, whereas acetonitrile and lithium perchlorate (>95.0%) were purchased from J.T. Baker and Aldrich, respectively. De-ionized water (DIW) was used throughout the experiments. All electrochemical experiments, including both potentiometric and amperometric measurements, were performed with a potentiostat/galvanostat (Autolab, model PGSTAT30, Utrecht, the Netherlands).

### 3.2. Preparation of Cholesterol Solution

The background electrolyte contains 0.02 M phosphate buffer, 10% (v/v) Triton^®^ X-100, 0.3 mM ferrocene and 0.1 M KCl, and cholesterol was added to the background electrolyte to form the 8 mM cholesterol solution. Afterward, both the background electrolyte and cholesterol solution were put into a constant temperature water bath at 65 °C for 6 hours until they become clear. Then they were allowed to cool down to room temperature and stored at 4 °C when not in use. Triton^®^ X-100 was used as a non-ionic surfactant to enhance the cholesterol solubility in aqueous solution. Moreover, the surfactant also plays a role in stabilizing the activity of the cholesterol oxidase, as reported in the literature [17].

### 3.3. Fabrication of the Enzyme Electrode

Electropolymerization was carried out in a three-electrode cell, consisting of a platinum disc with 3.14 mm^2^ area as the working electrode, a Ag/Ag^+^ as the reference electrode and a platinum wire as the counter electrode. Prior to the polymerization, the working electrode was polished by alumina powders with the particle size of 0.05 μm and then ultrasonically cleaned in DIW. The organic electrolyte solution containing 0.5 M monomer, 3-TPAA and 0.1 M lithium perchlorate was dissolved in acetonitrile. The red-orange poly(3-TPAA) film was electrochemically deposited on the working electrode by applying a constant current of 1.5 mA (or 0.48 mA/mm^2^) for 100 s. Finally, the Pt/poly(3-TPAA) modified electrode was washed by DIW and dried in air. Three electrodes, which were prepared at different deposition times of 5, 30 and 100 s, were assigned as electrodes A, B and C, respectively. After electrodepositing the polymer film on the electrode surface, the enzyme was immobilized by the covalent attachment. Initially, the Pt/poly(3-TPAA) electrode was put in an aqueous solution containing 0.015 M EDC and 0.03 M NHS for 1.5 hours in order to activate the free carboxylic groups of the polymer, and then immediately placed into a 0.02 M phosphate buffer solution with 100 U/ml cholesterol oxidase to react for another 1.5 hours. During the above process, the peptide bonds formed between the activated carboxylic groups and the free amino groups of the enzyme, thus leading to the formation of the enzyme electrode, Pt/poly(3-TPAA)-ChO. The whole immobilization process was carried out at room temperature. The enzyme electrode was stored under dried condition at 4 °C when not in use.

### 3.4. Determination of the Sensing Potential and Performance Test

A three-electrode system was constructed for sensing cholesterol by the amperometric method. The working electrode, reference electrode and counter electrode are the enzyme-modified electrode of electrode C, Ag/AgCl/sat’d KCl electrode and platinum wire, respectively. In order to search for the suitable sensing potential, the linear sweeping voltammetry (LSV) method was performed with respect to the background electrolyte and the 8 mM cholesterol solution in potential ranging from 0.30 to 0.80 V at a low scan rate of 0.1 mV/s. All the solutions for the LSV experiments were de-oxygenated. Moreover, the net current, namely, the difference between the sensing current and the background current, was plotted as a function of the potential and the suitable sensing potential was chosen in the limiting current plateau obtained from the LSV curve. The working electrode potential of 0.70 V with a sampling time of 200 s was chosen to obtain the steady-state sensing current. Furthermore, a calibration curve was established with cholesterol concentration ranging from 0 to 8 mM. Additionally, the interference effects with respect to ascorbic acid and uric acid were studied during cholesterol sensing. For this, 0.10 mM ascorbic acid and 0.40 mM uric acid were independently added into the cholesterol solution to detect the additional currents in separate experiments.

## 4. Conclusions

In this work, the amino groups of the cholesterol oxidase were successfully linked with the carboxylic groups of the Pt/poly(3-TPAA) activated by the two reagents, EDC and NHS, as confirmed by the FTIR spectra, SEM morphologies and EIS analyses. In addition, the cholesterol biosensor provides good performance characteristics. The linear range lies within the normal range of the human body (between 0 and 8 mM). The sensitivity of electrode B and response time (t_95_) are 4.49 mA M^−1^ cm^−2^ and 70 ∼ 90 s, respectively, and these values are acceptable when compared with the performance reported in the literature. Based on the signal to noise ratio of 3, the limit of detection is about 0.42 mM, which is a little bit higher than the values reported elsewhere. As for the selectivity, common interferences such as ascorbic acid and uric acid increase the original current at 5.2% and 10.3%, respectively. Most importantly, the biosensor possesses a good long-term stability. The sensing current still retains 88% of its original current even after 13 days.

## Figures and Tables

**Table 1.** The performance parameters of cholesterol biosensors reported in the literatures.

**Enzyme Electrode**	**Linear range (mM)**	**SEN (mAcm^−2^M^−1^)**	**LOD (M)**	**Response time (s)**	**Stability (days)**	**[Ref.]**
Pt/P(HEMA)^1^/PPy^2^-ChO	0.5–15	0.02	120	30	>360 (75%)	[7]
GC/TEOS^3^ sol-gel/HRP/ChO	2–10	0.48	-	-	-	[17]
Pt/PPy-ChO/o-PPD^4^	0–0.3	50.62	1.35	7.5	16 (70%)	[18]
Pt/TMOS^5^ sol-gel/ChOx/p(DB)^6^	0.06–3	0.58	-	51	32 (50%)	[19]
Pt/PAn^7^/ChO	0.01–0.1	2.22	-	-	-	[8]
Au/AET^8^+TP^9^/MP-11^10^/ChO	0.2–3	0.09	-	<20	-	[20]
Pt/Pt/PPy/ChO	0–0.4	1.1	14	6.3	>35 (70%)	[21]
W/ferrocyanide/[ChO/ChEt]	0.05–3	-	10	30	-	[22]
CPE/HMF^11^/POD/[ChO/ChEt]	0.001–0.15	9.5	-	-	-	[23]
GC/PPy/laponite/[ChO/ChEt]	0–0.025	13.2	0.5	50	10 (70%)	[24]

**Pt/Poly(3-TPAA)/ChO**	**0–8**	**4.49**	**420**	**70 ∼ 90**	**>13 (88%)**	**This work**

1 P(HEMA): poly(2-hydroethyl methacrylate),

2 PPy: polypyrrole,

3 TEOS: tetraethyl orthosilicate,

4 o-PPD: poly(o-phenylenediamine),

5 TMOS: tetramethoxysilane,

6 P(DB): poly(1,2-diaminobenzene),

7 PAn: polyaniline,

8 AET: 2-aminoethanethiol,

9 TP: 3-thiopropanol,

10 MP-11: microperoxidase-11,

11 HMF: hydroxymethylferrocene
